# Dataset of cone opponent mechanisms and spectral tuning in non-primate vertebrates

**DOI:** 10.1016/j.dib.2024.111166

**Published:** 2024-11-27

**Authors:** Carlay L. Teed, Samuel Hartzler, Esteban Fernández-Juricic

**Affiliations:** Purdue University, 610 Purdue Mall, West Lafayette, IN 47907, United States

**Keywords:** Colour vision, Retinal mechanisms, Comparative physiology, Photoreceptor

## Abstract

Cone and spectral opponency are fundamental to colour processing in many species and are well studied in primates. The data required to make interspecific comparisons of the neural mechanisms associated with colour processing is spread across a broad body of literature reaching back to the 1950ʼs across four retinal cell types and multiple brain regions. We aimed to produce a comprehensive dataset of all known cone opponent cells in non-primate vertebrates in image forming visual pathways. We completed a systematic literature search of two databases, Web of Science and Scopus, following PRISMA guidelines. From the data collected, we produced three datasets. One dataset contains cone opponency data that indicates which photoreceptors drive cell light responses. The second dataset contains spectral opponency data that represents the cell electrical responses to different wavelengths of light. Additionally, we developed a third database of photoreceptor data for the species for which cone or spectral opponency was reported to supplement the first two. These datasets will provide a synthesis of the data in the field of colour processing, can be used for interspecific and intraspecific meta-analyses, and can provide a starting point for understanding neural mechanisms behind wavelength comparisons in non-primate vertebrates.

Specifications Table

**Table:** 

Subject	Sensory Systems
Specific subject area	Neural mechanisms of colour vision
Type of data	Tables (.csv and .xlsx format) and Images. Raw, Means (± SD), Additional synthesis variables created by consolidating raw data.
Data collection	A Boolean search string, designed with the help of a librarian, was used to query Scopus and Web of Science databases. The combined queries identified 1723 studies, which were evaluated to identify studies with cone opponency data. Seventy-three studies were included, and one round of citation chasing based on these 73 studies identified 4977 additional studies (3485 after duplicates were removed) to review. After evaluation, 47 of those studies were also determined to have cone opponent information. Cone opponent data were harvested from the tables, figures, and text of these studies. Spectral opponent data were harvested from all studies that had been retrieved.
Data source location	Searches in English yielded global data that were harvested from sources published in English, Greek, Japanese, German, Korean, and Russian.
Data accessibility	Repository name: Open Science Framework Data identification number: DOI 10.17605/OSF.IO/45BNP Direct URL to data: https://osf.io/45bnp/
Related research article	None

## 1 Value of the Data

• These data entail a comprehensive collection of studies on cone opponent cellular mechanisms and spectral opponency in non-primate vertebrates.
• The cone and spectrally opponent literature is fragmented and challenging to synthesize as the data have been obtained from a diverse array of experimental methods and across decades of research dating back to 1953. Here, we have collected the studies that provide these data and extracted the specific elements (cone inputs and/or wavelengths of light the cell responded to) needed to classify and describe cone and spectrally opponent cells from text, tables and figures. While some studies reported the exact information needed, others required some manual processing of the reported data (i.e., quantification of the cell spectral responses, interpretation of retinal circuit maps) to enable the reader to draw direct comparisons between studies.
• These data can be used to identify gaps in our knowledge; for instance, determining cell types or major vertebrate classes that are underrepresented in existing studies, and generate hypotheses about the ecological and behavioural relevance of colour vision in different species. The multiple datasets can be used for studies on the evolution of visual mechanisms in vertebrates, phylogenetic analyses of visual traits, and comparative studies on cone and spectral opponency.
• Null points, a measure of the transitional wavelength between excitatory to inhibitory responses in spectral opponency, are made available for every cone opponent cell for which they were reported [1]. These data can be used to correlate the spectral properties of a cell with its cone inputs.

## 2 Background

Cone opponency is a mechanism by which colour is processed, whereas spectral opponency is the direct product of that processing. Cone opponent neural circuits receive input from multiple photoreceptors, typically cones which produce excitatory and inhibitory responses. Spectral opponency is evidence of cone opponency and indicates the magnitude and polarity of the cell responses to wavelengths of light [2]. Our objective was to collect every known occurrence of cone opponency in non-primate vertebrates and quantify the cone inputs into the cone opponent circuitry. If spectral opponency was uncovered in our search, we also digitized and computed the cell spectral properties.

## 3 Data Description

**Fig. 1:**
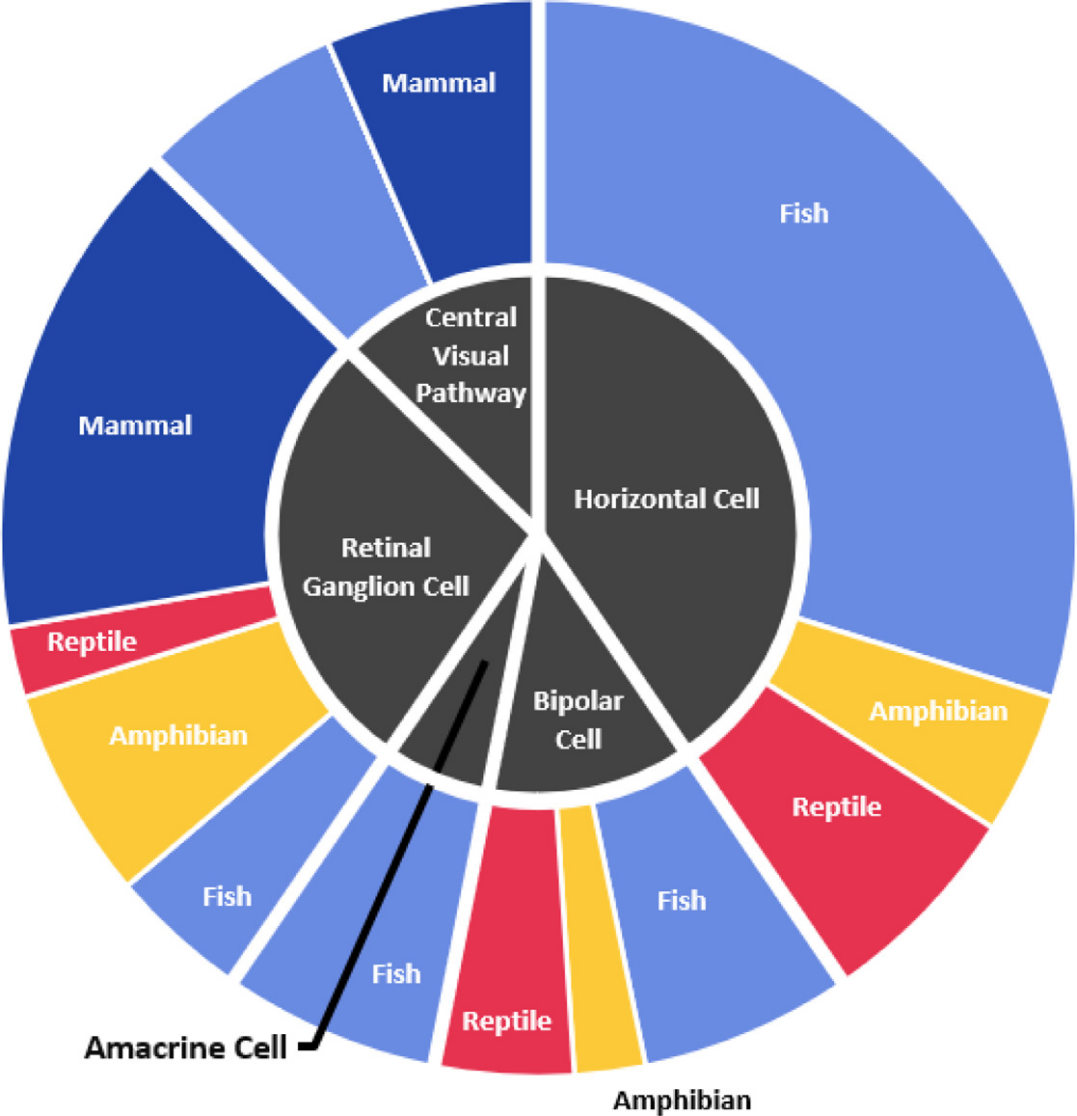
The proportion of species found to express cone opponency in different cell types and the major vertebrate classes to which those species belong. Of the 16 species reported for horizontal cells, 11 species were fish. Of the 13 species reported for Retinal Ganglion Cells, 7 were mammals.
2) Spectral Opponency Excel File
  a. Spectral opponency is an electrophysiological phenomenon that can be recorded from cone opponent cells. Spectrally opponent cells respond with excitation to some wavelengths of light, and inhibition to others. For all spectrally opponent cells, their responses to at a minimum of two wavelengths have been reported. In this data file, we include taxon information, the specific wavelengths tested, the cell response to those wavelengths, the location within the visual system, a classification of the cell response (e.g. biphasic, triphasic, or tetraphasic, see spectral opponency metadata for definitions and examples), spatial properties (if known), the source figure from which we extracted the data, and the source of the data.
  b. Fig. 2 displays the proportion of species which were represented in spectral opponency studies in different cell layers. Fig. 2 also breaks down the proportion of species which fall into each major vertebrate classification for each cell type. Fish made up the majority of species studied for every cell type reported except for the cells in the central visual pathway, where mammals made up the majority of reported cells.

1) Cone Opponency Excel File
  a. Cone opponent cells are neurons in the image forming pathway of the visual system that receive excitatory input from one or more photoreceptors, and inhibitory input from one or more other photoreceptors (usually cones). Every cell is defined by their photoreceptor inputs, but cone opponent cells are found throughout the visual system (retina and central visual processing areas.) and have diverse spatial properties (i.e., some cells have the same photoreceptor inputs throughout their receptive fields, others have a center-surround organization where the cones that drive their center response differ from those that drive the cell response in the periphery). In this file, we have collected every occurrence of cone opponent data reported in our systematic review of the literature. For each cone opponent cell, we specify taxon information, location within the visual system, spatial properties if known, number of cells sampled, which types of photoreceptors connect to the cone opponent cell (i.e., overall sensitivity of the photoreceptor), cell null point(s), which are the wavelength(s) at which the cell spectral response is neither positive nor negative), which photoreceptors are excitatory or inhibitory, and the source of the data.
  b. Fig. 1 displays the proportion of species which were represented in cone opponency studies in different cell layers. Fig. 1 also breaks down the proportion of species which fall into major vertebrate classes for each cell type. Fish were represented in the majority of studies in all areas studied, except the Retinal Ganglion Cell Layer, where the majority of species studied were mammals.

**Fig. 2:**
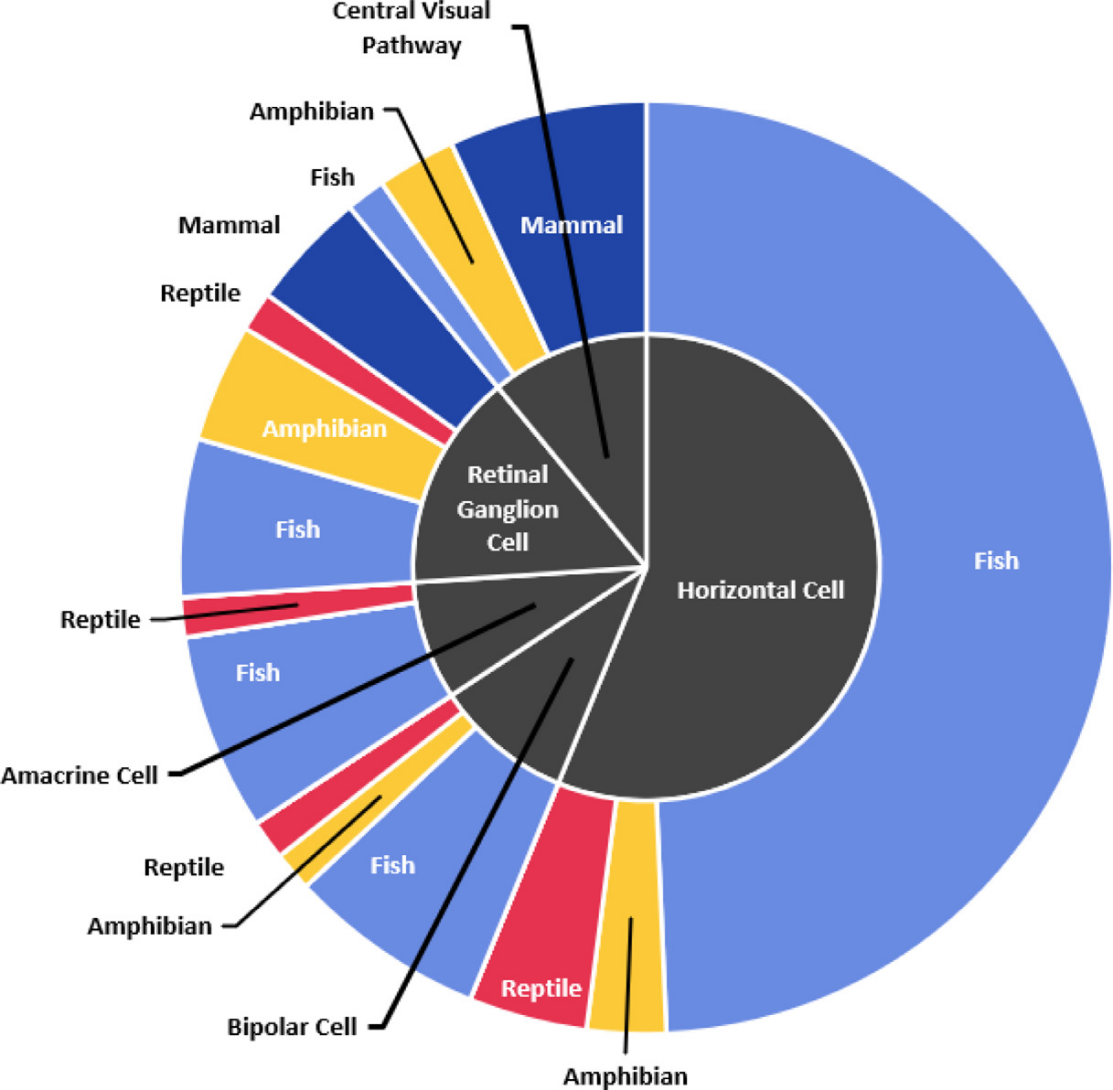
The proportion of species found to express spectral opponency across cell types and the major vertebrate classes to which those species belong. Of the 46 species reported for horizontal cells, 41 species were fish.
3) Photoreceptor Properties Excel File
  a. This file contains the number, sensitivity, some morphological traits of photoreceptors (single or double or twin, rod or cone), and taxonomy data for each species that cone opponency is known (i.e., cone opponency file). These data may be used to enhance our understanding of cone opponent colour vision mechanisms by providing more information about which types of photoreceptors (i.e., long-, short-, etc. wavelength sensitive) are in the circuit.
4) Source List
  a. This Excel file contains the citation information for each study used to collect information on cone opponency, spectral opponency, and photoreceptor properties. Each source has a unique ID, by which it is referenced in the Cone Opponency, Spectral Opponency, or Photoreceptor Properties excel files. This file indicates which excel files the source was used in (there may be more than one), the title, authors, publication year, journal, volume, issue, page number, the species used, and additional notes on the study.
5) Null Images Folder
  a. This folder contains the images extracted from the studies that were used to quantify a cone opponent cell null point(s) (i.e., the wavelength(s) at which the cell spectral response is neither positive nor negative), if the null point was not specified in the text of the study. These images are organized by location within the visual system. The null points were extracted using the package digitize [3]. The R code used to quantify the null point is included in this folder.
6) Spectral Images Folder
  a. This folder contains the images extracted from the studies that were used to quantify spectral opponency and the R code used to extract the data from these figures. The spectrally opponency information was extracted using the R package metaDigitise [4].

## 4 Experimental Design, Materials and Methods

**Fig. 3:**
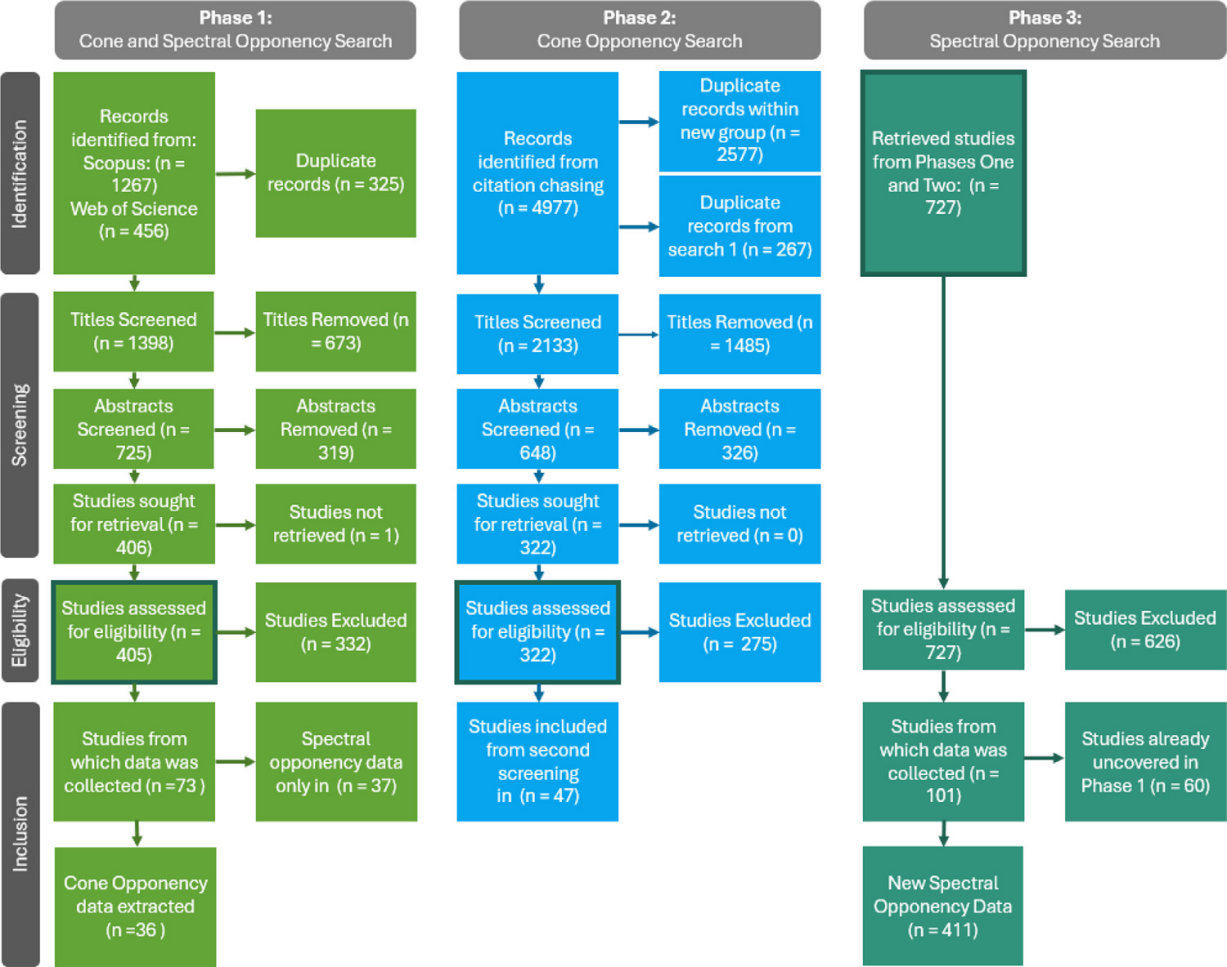
Reproducible Literature Search Flow Chart: Our reproducible literature search was conducted in three phases. The first phase, aimed at identifying and including studies about cone opponency in non-primate vertebrates, uncovered cone and spectrally opponent studies. The first search was insufficient for identifying every study on cone opponency in non-primate vertebrates. To expand our dataset, we used citation chasing in the second phase. This phase identified many more cone opponent studies. Our last phase was targeted at identifying spectrally opponent studies which may or may not have information about cone opponency.
  1.2 Phase One Identification: To collect sources for this review, we engaged in reproducible literature search following PRISMA guidelines [5]. The reproducible literature search began when we first familiarized ourselves with the literature using a key review (Twig et al. 2003), from which we generated our search terms. CLT consulted with a Purdue University librarian to generate search queries (detailed in Table 1) for two separate databases, Scopus and Web of Science all databases. Scopus returned 1267 results and Web of Science returned 456 results (Fig. 3). Between the two databases we started with 1723 studies. Initially, duplicate titles were removed using the remove duplicates function from Excel, removing 208 studies (Fig. 3, Phase 1 Identification). However, some titles had syntax discrepancies between databases (e.g., ganglion cell vs. ganglion-cell) and those studies were removed manually by sorting the titles alphabetically and reading through them. Altogether, there were 1398 unique studies to review (Fig. 3).

1. Experimental Design, Materials, Methods:
  1.1 The process of identifying potential studies, screening them for relevant data, and including them in our dataset took place across three phases (Fig. 3). The first phase used a Boolean search string designed to identify studies with data on cone opponency. The second phase expanded upon our potential pool of studies by using citation chasing based on the included studies from the first phase. The third phase was geared towards identifying and extracting spectrally opponent information from all the studies that were retrieved after having passed the cone opponency screening.

**Table 1:** Database search screens and search dates for both databases searched, scopus and web of science.

**Scopus**	“TITLE-ABS-KEY ( c-type OR oppon* ) AND ( photoreceptor OR horizontal OR bipolar OR amacrine OR ganglion OR nucleus ) AND ( retina OR antagonistic OR null ) AND NOT ( "Vitamin C" OR "Vit C" )”	First Searched: 01/19/2022 Last searched: 01/19/2022
**Web of Science (Wos)**	“AB=((c-type OR oppon* ) AND ( photoreceptor OR horizontal OR bipolar OR amacrine OR ganglion OR nucleus ) AND ( retina OR antagonistic OR null )) OR TI =((c-type OR oppon* ) AND ( photoreceptor OR horizontal OR bipolar OR amacrine OR ganglion OR nucleus ) AND ( retina OR antagonistic OR null )) OR KP = ((c-type OR oppon* ) AND ( photoreceptor OR horizontal OR bipolar OR amacrine OR ganglion OR nucleus ) AND ( retina OR antagonistic OR null ))”	First Searched: 01/19/2022 Last Searched: 04/06/2023
  1.3 Phase One Screening: After removing duplicates, we reviewed all the titles for content, excluding any titles that did not match our goals. Titles were removed if they fit the exclusion criteria specified in Table 2. We read the abstracts of studies that had relevant titles (725 studies). Of those studies, 319 were irrelevant based on the abstract (Fig. 3, Phase 1 Screening), following our exclusion criteria (Table 2). The rest were either relevant or unclear. The process was conservative, choosing to retain more studies until the end (Fig. 3).

**Table 2:** Exclusion criteria for cone and spectral opponency screening (Fig. 1, Screening).

**Methodological constraints**	If the study exclusively collected data without using electrophysiological methods that would allow us to identify the cell type (examples Microspectrophotometry (MSP), electroretinogram (ERG), morphological studies, or psychophysics), we would not include the data.
**Injury or disease**	If the study investigated cellular function under diseased circumstances (e.g., diabetes), which will not indicate cone opponency in a healthy animal, we would not include the data.
**Non-visual organs and senses**	If the study was about organs not related to vision (e.g., kidney) or about non-visual senses (e.g., hearing), we would not include the data.
**Visual phenomena unrelated to colour vision**	If the study investigated achromatic visual phenomena such as direction sensitivity, blind sight, intrinsically photosensitive retinal ganglion cells, or night vision, we did not include the data.
**Molecular, cellular, development, or Genetics**	If the study was about the phototransduction cascade, opsin genetics, stem cells, or neurotransmitters, we did not include the data.
**Behaviour and evolution**	If the study was about how colour relates to behaviour and evolution, we did not include the data. Some examples are the effects of colour on sexual selection, animal coloration, and phototaxis.
  1.4 Phase One Eligibility: The remaining 406 studies were sought for retrieval (Fig. 3, Phase 1 Eligibility). Only one study could not be retrieved after consulting with Purdue University librarians. Four hundred and five studies were downloaded and stored in a lab drive, or physical copies were mailed to us through Interlibrary loan if a digital format was inaccessible. We developed eligibility criteria with five components. We only accepted studies that (1) had electrophysiology data, (2) recorded cellular responses to at least two wavelengths of light, (3) could identify the cell type they were recording from, (4) collected data from non-primate vertebrates, and (5) reported the genus and species name of the animal studied, except in cases where the common name may only refer to one species (e.g. Belgian Hare). Three hundred and thirty-two studies were rejected for failing to meet these eligibility criteria. Seventy-three studies were carefully evaluated for cone opponency and spectral opponency. Of those 73 studies, 36 were identified to contain cone opponency data (13 cone opponency only, 24 cone and spectral opponency), and 37 were identified to contain only spectral opponency data (Fig. 3, Phase 1 Inclusion).
  1.5 Phase One Cone Opponency Inclusion: Cone opponency data was extracted from the text, tables, or figures from 73 studies. We determine that cone opponency data was able to be included if:
    1) At least one cell described has excitatory and inhibitory inputs from different photoreceptors. These photoreceptor inputs may be in the same region of the receptive field (e.g. L+M- in the entire receptive field or L+M- center/L+ surround) or they may be in different regions of the receptive fields (eg. L+ center/M- Surround, or L+M+ center/M- surround).
    2) The authors specified the cone mechanism that drives the spectral responses.
    Studies were excluded, however, if they fit any of the following exclusion criteria:
    1) The authors did not specify the cone inputs to any opponent cells.
    2) The authors did not specify the polarity of the cone input (e.g., is the L cone driving the excitatory or inhibitory response?)
    3) The authors concluded with uncertainty about the cone connections. (e.g., “We think the S cone is involved but we are not sure.” Or “the green response might come from either the M cone or the rod.”)
    4) The authors did not report the null points for horizontal cells.
    5) Authors used the spectral opponency data as evidence for the existence of cone classes, and no data were available on the cones in that species.
    6) Authors were unsure of the cell type being recorded from (i.e. Horizontal cell, Ganglion cell, etc.).
    7) The authors only reported input from one cone class.
    8) Opponency was only visible under adapting light, and not during white light or dark adaptation.
    9) The scanned study file was corrupted, and we could not extract the necessary information, nor could we obtain a physical copy.
    There are variations in naming conventions for cone classes based on their spectral sensitivity. For our database we chose to report cone inputs to opponent cells using UV, S, M, & L as abbreviations for Ultraviolet Sensitive, Short-Wavelength Sensitive, Mid-Wavelength Sensitive, and Long-Wavelength Sensitive, respectively. Inputs from rods are labelled as Rod 1 and Rod 2 (if two rods are involved in opponency) and inputs from Double/Twin cones are all abbreviated as DC. If the primary or accessory member of the double cone was specified in the study, we specified it in the table. Data reporting methods for horizontal cells differed from all other cell types. Horizontal cells are the first direct contacts of the photoreceptors, and as such their inputs are easier to determine and simpler than other cell types. For horizontal cells, we reported if each photoreceptor input was excitatory or inhibitory and we did not report any spatial properties as these cells always have the same inputs regardless of position within the cell receptive field [6]. Additionally, horizontal cells only have on responses, meaning that they respond when a light turns On, and do not produce a response when a light turns off [7]. For all other cell types, we reported spatial properties (are the receptive field inputs uniform, or does it have a centre-surround arrangement?) and if the photoreceptor input was “On” or “Off”.

1.6 Phase One Spectral Opponency Inclusion: Studies that met all eligibility criteria and reported the cell response to light were included in our spectral opponency dataset. Spectral opponency was extracted from figures, tables, and directly from the text of 70 studies (37 only cone opponency, 23 cone and spectral opponency). Spectral opponency data was identified as the cell response (e.g. change in firing rate or change in membrane potential) to a specific wavelength of light. This response was always compared to a baseline, which could either be its resting membrane potential or basal firing rate. We prioritized extracting spectral opponency data from figures, which typically had the most data and indicated the wavelengths to which a cell responded, and the degree of cell response to each wavelength. Data from figures was extracted using the R package metaDigitise [4]. Depending on the type of figure reported, some raw values (43% of the figures digitized) required additional processing after extraction to be comparable across studies (methods for additional processing can be found in the spectral opponency metadata). If figures were not available, we extracted spectral opponency data from tables, which indicated the polarity of cell response to a light but may have only described the degree of cell response on a qualitative scale (if cell inputs were indicated with + or – symbols, we converted them to numeric 1 and -1). Lastly, if there were no tables or figures, rarely the spectral opponency data could be obtained from the text. Typically, the text only contained peak wavelengths. In this case, we extracted exactly what was described in the text (e.g., the cell responded maximally with excitation at 550 nm and inhibition at 475 nm). After collection, cell responses were normalized so that their peak response is 1 (or -1 if their peak response is inhibition) and response at their null point (defined as the wavelength where the cell gives no response) is 0. We deemed this normalization necessary to allow for comparisons across studies.
1.7 Phase Two Identification, Citation Chasing: after reviewing all studies that were included, it became apparent that some sources referenced by the studies we included had not appeared in our initial search. To fill in the gaps in our dataset, we engaged in a round of citation chasing. We used Web of Science to identify all studies that cited the study we had collected data from (n = 73), and all that the included studies were cited by (Fig. 3 Phase 2, Identification). We identified 4977 studies. Duplicates were removed from the second batch of studies following the same procedure as described in 4.2, and 2577 duplicates were identified and removed. We then searched for duplicates of studies that we found in the first literature search (4.2), and 267 studies were removed (Fig. 3, Phase 2 Identification).
1.8 Phase Two Screening: we screened the remaining titles (n = 2133) for relevance, and removed 1485 titles (Fig. 3, Phase 2 Screening). Of the 648 remaining studies, abstracts were screened for relevance following the same exclusion criteria as in phase one (Table 2), and 326 studies were removed.
1.9 Phase Two Eligibility: Three hundred and twenty-two studies were downloaded or physically obtained. These studies were reviewed using the same inclusion criteria as the previously reviewed studies (4.3). We only accepted studies that (1) had electrophysiology data, (2) recorded cellular responses to at least two wavelengths of light, (3) could identify the cell type they were recording from, (4) collected data from non-primate vertebrates, and (5) reported the genus and species name of the animal studied, except in cases where the common name referred to one species (e.g. Belgian Hare). In this search effort, we exclusively identified studies that had cone opponency data. Spectral opponency was not reviewed at this step.
1.10 Phase Two Inclusion: Forty-seven additional studies were identified to have cone opponency data and were included in the second round. Data were collected from figures, text, and tables as was done in 4.5.
1.11 Phase 3, Spectral Opponency Identification: to assess studies for spectral opponency, we reviewed the 727 studies (Phase 3, Identification) that were retrieved from the two searches (405 studies from phase one (Fig. 3, Phase 1 Eligibility), 332 studies from phase two (Fig. 3, Phase 2 Eligibility)).
1.12 Phase 3, Spectral Opponency Eligibility: because spectral opponency is an electrical property that is recorded from cells exposed to different wavelengths of light, when screening studies for spectral opponency, we had 4 criteria for inclusion: the study (1) used electrophysiological methods to measure the response of a cell, (2) measured two or more wavelengths of light, (3) the specific wavelengths of light needed to be reported either in a figure or in the text, and (4) the species taxonomic name (genus and species) was reported (i.e., use of common species names was not sufficient unless there is only one animal that can be referenced by that common name; such as , domestic cat). When multiple species were studied, the study must have specified which electrical recordings were made from which species to be included. Six hundred and twenty-six studies were excluded for not meeting these criteria.
1.13 Phase 3 Spectral Opponency Inclusion: Forty-one additional studies were found to contain spectral opponency data, bringing the total to 101 (Fig. 3, Phase 3: Inclusion). Data were digitized and extracted as described in 4.6.
1.14 Photoreceptor Dataset: For all species for which we know cone opponency data, we sought out data about photoreceptor properties. Ideally, we wanted to include how many photoreceptors (rods, cones, and double/twin cones), and the peak sensitivities of those photoreceptors for each species, which can be informative for studies on biokinetics. Initially, we checked each study with cone opponency data for information about the photoreceptor peak sensitivities, and if they were reported in the study, we used the data provided in it. If, instead, the study only indicated the number of cones or gave an imprecise classification of the cone sensitivities (e.g., L for long wavelength sensitive or R for red cone), we searched Google, Web of Science, and Google Scholar using a combination of the search terms [Species Name], photoreceptors, MSP, Microspectrophotometry, opsin, and spectral sensitivity. If a study was found that provided the number of photoreceptors (including rods and double cones, if present) and their peak sensitivities, we included that study in our search. If the search only returned studies with partial data (e.g., number of rods and cones in one study, and peak sensitivity of cones in a separate study) multiple studies were included for one species. Not all data was available for all species.

## Limitations

1) The spectral opponency search is not comprehensive. We only recorded spectral opponency from studies that were screened for cone opponency.
2) The original search queries were only used in January of 2022. Any study about cone opponency published after this date would have only been included if they cited one of the original 73 accepted studies due to our citation chasing approach for our second search.
3) Evidence of cone opponency was discernible in other studies which turned up in our search, but they did not report enough data to meet the criteria for inclusion. (e.g., the authors reported the cone inputs, but did not report if the cones drove the excitatory or inhibitory response)
4) We excluded all instances of cone and spectral opponency that were not part of the image forming visual pathway. For instance, cells in the pineal gland of fish [8] and parietal eye of lizards [9] also exhibit spectrally opponent responses when recorded from but are not involved in colour vision.

## Ethics Statement

The authors have read and follow the ethical requirements for publication in Data in Brief and confirming that the current work does not involve human subjects, animal experiments, or any data collected from social media platforms.

## CRediT authorship contribution statement

**Carlay L. Teed:** Conceptualization, Methodology, Software, Validation, Investigation, Data curation, Writing – original draft, Visualization. **Samuel Hartzler:** Methodology, Investigation, Data curation, Writing – review & editing. **Esteban Fernández-Juricic:** Conceptualization, Methodology, Writing – review & editing, Supervision.

## Data Availability

OSFDatabase on Cone and Spectrally Opponent Cells in Non-Primate Vertebrates (Reference data).
